# Information for the People

**Published:** 1864-07

**Authors:** W. H. Atkinson


### "INFORMATION FOR THE PEOPLE."

Read before the Brooklyn Dental Association, April 13, 1864.

BY W. H. ATKINSON.

When we speak of the people in contradistinction to the profession, ideally there is a well-marked distinction; but when we face the naked facts of the case, we will find that many whom we designate as the people are in fact much better posted in the principles upon which beneficent practice should be based in *all* cases than those who essay to make the application for the use and behoof of the people.

What, then, is *information?* It is nothing more nor less than the *forming within* the mental receptacles clear conceptions of the various principles of knowledges essential to full comprehension of organic being, in the various acts appropriative and distributive appertaining to health and disease; and the intelligent ability to assist, retard or supplement these various acts and functions without detriment to the organism which we prescribe for or manipulate.

If this be the only legitimate definition to the term, who of us all, of profession or people, can truly be said to be *informed?* In the complete sense, no one; in the approximative sense, there will be a comparative equality of profession and people, mutually *informed* or deplorably *uninformed.* How shall we determine this? Simply by a few tests.

First, then, I ask, where do we meet the greater proportion of patients who are well-informed, and have a just apprehension of what they ought to expect and require at the hands of the dentist? We would naturally hope to find medical men as a class in the possession of this knowledge, but unpleasant as may be the reflection, they are, as a class, the last to properly estimate and appropriate the knowledge and skill of the intelligent dentist.

Were we to rigidly scrutinize the causes of this, it would not increase either our respect for medical science or medical men—the former being so fractional and indefinite; the latter so lackadaisical and taciturn, diffusive or contracted as to repel the ardor or patience to adequately measure science or men.

The mass of mind popular or professional is more disposed to follow than take the responsibility and arduous labor to lead. Hence the paramount importance of having the leaders quite right in all positions assumed where the principles are not readily demonstrable to all. Pernicious doctrines and practices are more frequently incident upon *misapprehension* on the part of those who follow, than the *doctrines* enunciated by those who lead.

First, let the dental profession be thoroughly indoctrinated and familiarized with all known diseases and remedies, conversationally scrutinized by our sharpest and most persistent conjoined ability, correctly recorded and codified, and we shall have a basis of institutes upon which to stand firmly, to instruct the people what to know, and what to do to secure them against mischief, or remedy this so far as in our power, when it has found lodgment, local or systematic, involving discomfort or danger.

The nutrition, physiological and pathological of the mind, is precisely like these same actions occurring in the habitat and symbol of the ghost in the body, in general, or in the molecule in singular and particular, dependent upon equivalency of receptive, appropriative and rejective forces in the normal, and disturbance of this balance in the abnormal activities. Hence the propriety and imperative necessity for profession and patient to understand the importance of compatibility and incompatibility to each other, and the consequence of both resultant upon the close intimacy of the entire being in making the operations within the sacred temple of the human sentiency. For where mutual compatibility exists both are benefited by the contact, intensifying the molecular diastole and systole, enlarging the force and quantum of the life currents in the unseen departments of organic being.

But where the compatibility is confined to the receptivity of one and impartation from the other, the physiological currental flow is converted into pathological expression, and one suffers robbery to endow the other with the true riches of life and being.

Cases of incompatibility of both or one is of too common occurrence to require the enumeration of special examples. Where it obtains on the part of each the shock of the ghost is immediate and repellant in the degree of the intensities of the two constitutions added together. In such cases good service can not result from the forced operation if, from a false etiquette or other cause, nature be disregarded, and the abortive effort be made. Affection and defection, purely or conjointly, are always consequent upon the proximity of personalities necessary to the execution of an operation.

Faithfully and skillfully performed, dental and surgical service is the most concentrated of our labors, and hence intolerably exhaustive where incompatibility and defection hold the dominion to the exclusion of compatibility and affectionate interest which should blend in all our works.

He who is always surcharged with the dionizing life presence is alone able to spare the out-go to do good service to the wicked or the extremely weak in mind and body. All feeling and consequently all suffering occurs in the ghost, and not in what is called the physical body. Large sluggish bodies may be literally cut to pieces without pain to considerable degree, because of the immensity of size of the tenement compared to the tenant.

Fine, thin, small personality is pained at the simple presence of an incompatible coarse person, because the ghost is so much larger than its tenement that it pervades the atmosphere for considerable space beyond the limit of the body, and is in its antennæ unprotected by fleshy covering.

When, from sufficient cause, the teeth become inflamed, sensitive, tender, we may be sure that the sentiency projects beyond the normal limit, and thus suffers from various ethereal and corporeal contacts; incompatible ethers being more painful than the coarsest and hardest organic solids, the latter having an obtunding, the former an intensifying effect.

As before hinted individuals may be compatible in one state of conditions and quite the reverse in states of diversity of relative standing. So it is a matter of no easy attainment to decide at once in all cases upon the ultimate or alternate compatibility and incompatibility, spiritually, mentally and physically, of persons of our own or opposite sex. In fact, in just so far as our own sex are compatible to us they are relatively in the opposite condition of molecular and systemic polarity. Now to work this *information* serially up from the crudest organic physical basis of functional apprehension, in a demonstration of all its consecutive steps, it demands great desire and earnestness to know on the part of the learner, and great patience and untiring devotion to the cause of humanity, science and art on the part of him who has wrought it out by the inspiration of his own necessities and love of the race. But nothing short of this sort of devotion, heartily entered into by our body, can ever adequately investigate, understand and promulgate complete understanding of the organism in its healthy and unhealthy efforts to sustain individual life and being.

This is indeed a tremendous "experimentum crucis," through which to pass our infant body into the resplendent temple of demonstrative science of life, properly so called; but let the conception once deeply settle its purpose to know, upon us as a whole, and the prophesy will not have to wait long for brilliant and satisfactory fulfillment.

Let us, my dear fellow-apostles of dental science, set ourselves earnestly down to the work to know and then communicate the principles and practice of our beneficent mission first to our brethren of the profession, and then to the people in charge of the dear coming citizens of the community that is to be, in which shall dwell spiritual, mental and corporeal righteousness.

